# Effectiveness of Bachmann's Bundle Pacing in a Patient With Severe Heart Failure and Refractory Atrial Fibrillation

**DOI:** 10.1002/joa3.70466

**Published:** 2026-09-24

**Authors:** Yosuke Murase, Yasuhiro Ogawa, Hajime Imai, Keita Mamiya, Katsuhiro Kawaguchi

**Affiliations:** ^1^ Department of Cardiology Komaki City Hospital Komaki Japan

**Keywords:** atrial fibrillation, Bachman's bundle pacing, cardiac resynchronization therapy, heart failure, left atrial strain

## Abstract

This case report highlights CRT‐D with Bachmann's bundle pacing (BBP) in a patient with severe heart failure and refractory atrial fibrillation. BBP preserved atrioventricular conduction without PR prolongation, maintained left ventricular–only pacing, and was associated with suppression of atrial fibrillation and improvement in left atrial contractile function.

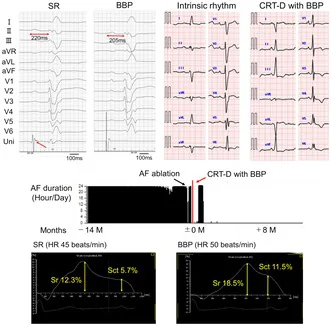

Bachmann's bundle pacing (BBP) has been reported to suppress atrial fibrillation (AF) [1]. However, its efficacy in patients with advanced heart failure undergoing cardiac resynchronization therapy (CRT) remains unclear.

A 71‐year‐old man with long‐standing persistent AF and chronic heart failure due to ischemic cardiomyopathy who had previously undergone implantation of a single‐lead implantable cardioverter‐defibrillator (ICD) had been followed at our outpatient clinic. He was admitted because of worsening exertional dyspnea (New York Heart Association [NYHA] functional Class IV). Laboratory tests showed elevated brain natriuretic peptide (BNP) at 1154.9 pg/mL and serum creatinine at 2.5 mg/dL. He received optimized guideline‐directed medical therapy for heart failure with reduced ejection fraction: bisoprolol 5 mg/day, sacubitril/valsartan 200 mg/day, empagliflozin 10 mg/day, and eplerenone 25 mg/day. Despite this optimized medical therapy, transthoracic echocardiography on admission demonstrated a left ventricular ejection fraction (LVEF) of 12%, left ventricular end‐diastolic volume index (LVEDVI) of 146 mL/m^2^, left ventricular end‐systolic volume index (LVESVI) of 127 mL/m^2^ and left atrial volume index (LAVI) of 72.8 mL/m^2^, with moderate mitral regurgitation. Mechanical LV dyssynchrony could not be clearly assessed on echocardiography because of the severely impaired LV systolic function. Despite diuretic therapy, heart failure did not improve sufficiently. For rhythm control, pulmonary vein isolation and posterior wall isolation were performed using a pentaspline pulsed field ablation catheter. A 12‐lead electrocardiogram obtained immediately after ablation during sinus rhythm showed first‐degree atrioventricular (AV) block (PR interval, 220 ms), nonspecific intraventricular conduction delay (NICD) (QRS duration, 177 ms), and intra‐atrial conduction delay (Figure 1). Ventricular pacing was < 1% because the AV delay was programmed to allow intrinsic AV conduction. However, AF recurred shortly after discharge. Bepridil 100 mg/day was initiated, and electrical cardioversion was performed, but sinus rhythm could not be maintained. Although bepridil was subsequently replaced with amiodarone 200 mg/day, AF control remained poor. Persistent AF led to worsening heart failure, resulting in readmission. Electrical cardioversion was performed after readmission; however, the patient exhibited sinus bradycardia and marked QT prolongation, which resulted in frequent ICD shocks due to torsades de pointes.

**FIGURE 1:**
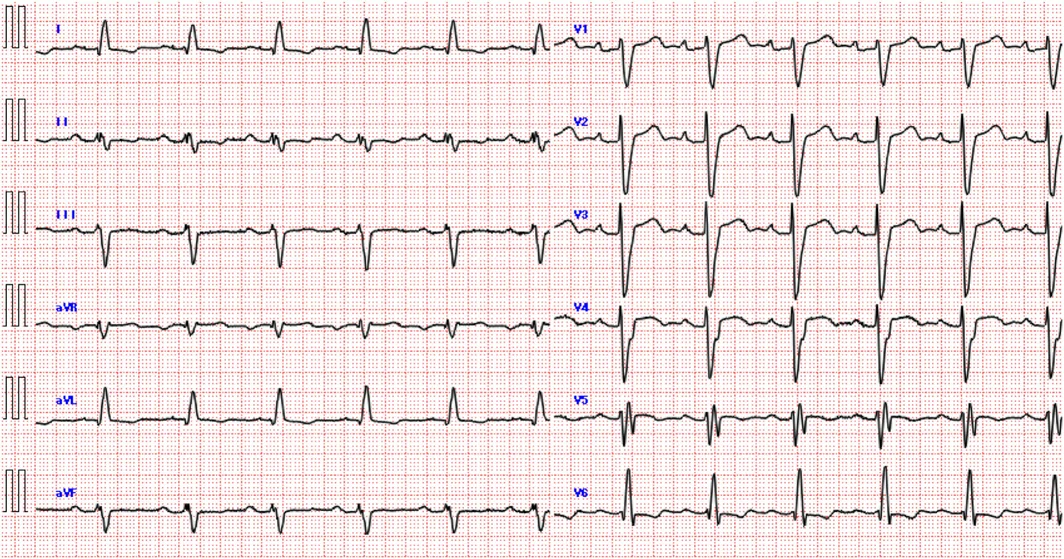
Twelve‐lead electrocardiogram before cardiac resynchronization therapy with defibrillator upgrade. Sinus rhythm was present with a heart rate of 71 beats/min. First‐degree atrioventricular block was observed (PR interval, 220 ms). The QRS duration was prolonged to 177 ms, consistent with nonspecific intraventricular conduction delay. The P‐wave duration was 138 ms, indicating intra‐atrial conduction delay.

Despite being within the post‐ablation blanking period, refractory AF, severe heart failure, and drug‐induced sinus bradycardia necessitated urgent rhythm control and atrial pacing. Therefore, cardiac resynchronization therapy defibrillator (CRT‐D) upgrade was performed to address both ventricular conduction delay and the need for atrial pacing. The AdaptResponse trial showed that maintaining a high percentage of left ventricular (LV)‐only pacing using the Adaptive CRT algorithm is associated with reduced risks of cardiac death and heart failure hospitalization [2]. However, because this patient had sinus bradycardia and first‐degree AV block, atrial pacing from the right atrial appendage (RAA) could prolong AV conduction and reduce the percentage of LV‐only pacing. Therefore, the atrial lead (SelectSecure 3830, Medtronic, Minneapolis, MN) was positioned at the high atrial septum using a delivery sheath (C315‐S5, Medtronic). Bachmann's bundle pacing (BBP) capture was confirmed by a paced P‐wave morphology showing higher‐amplitude P waves in the inferior leads and a P‐wave duration in lead II more than 10 ms shorter than that during sinus rhythm, consistent with the diagnostic criteria for BBP [3] (Figure 2). As shown in Figure 2A, the AV interval during BBP was not prolonged compared with that during sinus rhythm. The atrial lead also showed acceptable parameters (threshold 0.625 V/0.4 ms, P‐wave sensing 1.1 mV, impedance 760 Ω). The LV lead (Attain Stability Quad 4798, Medtronic) was positioned in the anterolateral coronary sinus branch because no other coronary sinus branches of suitable size were available for lead placement and this branch was located opposite and remote from the right ventricular lead. Within the anterolateral coronary sinus branch, the LV mid site (LV 2–3) showed the greatest electrical delay, with a QLV of 100 ms, and was therefore selected as the LV pacing site (Figure 3). Post‐implant electrocardiography showed QRS narrowing (Figure 2B). The pacing mode was set to DDD with a lower rate of 70 beats/min to maintain the atrial pacing percentage as high as possible. After the CRT‐D upgrade, guideline‐directed medical therapy for heart failure was continued unchanged, while amiodarone was discontinued and bepridil 100 mg/day was continued. After discharge, the atrial pacing percentage was nearly 100%, and the LV‐only pacing percentage reached approximately 80%.

**FIGURE 2:**
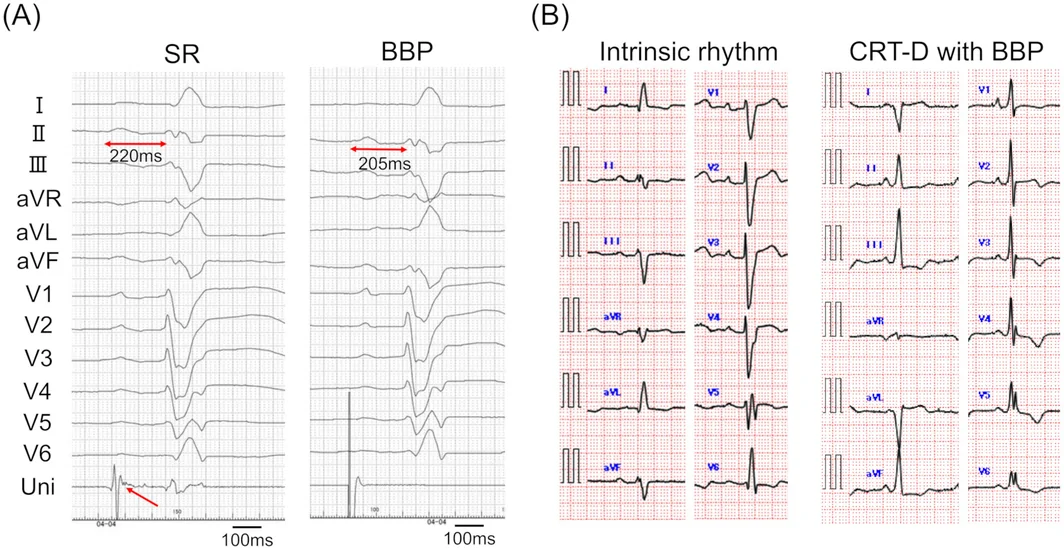
(A) Comparison of PR intervals during sinus rhythm (SR) and Bachmann's bundle pacing (BBP). The PR interval was 220 ms during sinus rhythm and 205 ms during BBP, indicating no prolongation of atrioventricular conduction with atrial pacing. Unipolar electrogram recorded at the BBP site during SR. A low‐amplitude, fragmented Bachmann's bundle potential (red arrow) was identified at the tip electrode in the middle portion of the P wave. (B) Comparison of twelve‐lead electrocardiograms during intrinsic rhythm and after cardiac resynchronization therapy with defibrillator upgrade with BBP. The QRS duration was shortened to 135 ms. The atrial pacing morphology showed positive P waves in leads I, II, III, and aVF, a biphasic P wave in lead V1, and higher‐amplitude P waves in the inferior leads compared with sinus rhythm. The P‐wave duration was shortened to 117 ms, more than 10 ms shorter than during sinus rhythm, fulfilling the diagnostic criteria for BBP. BBP, Bachmann's bundle pacing; CRT‐D, cardiac resynchronization therapy with defibrillator; SR, sinus rhythm.

**FIGURE 3:**
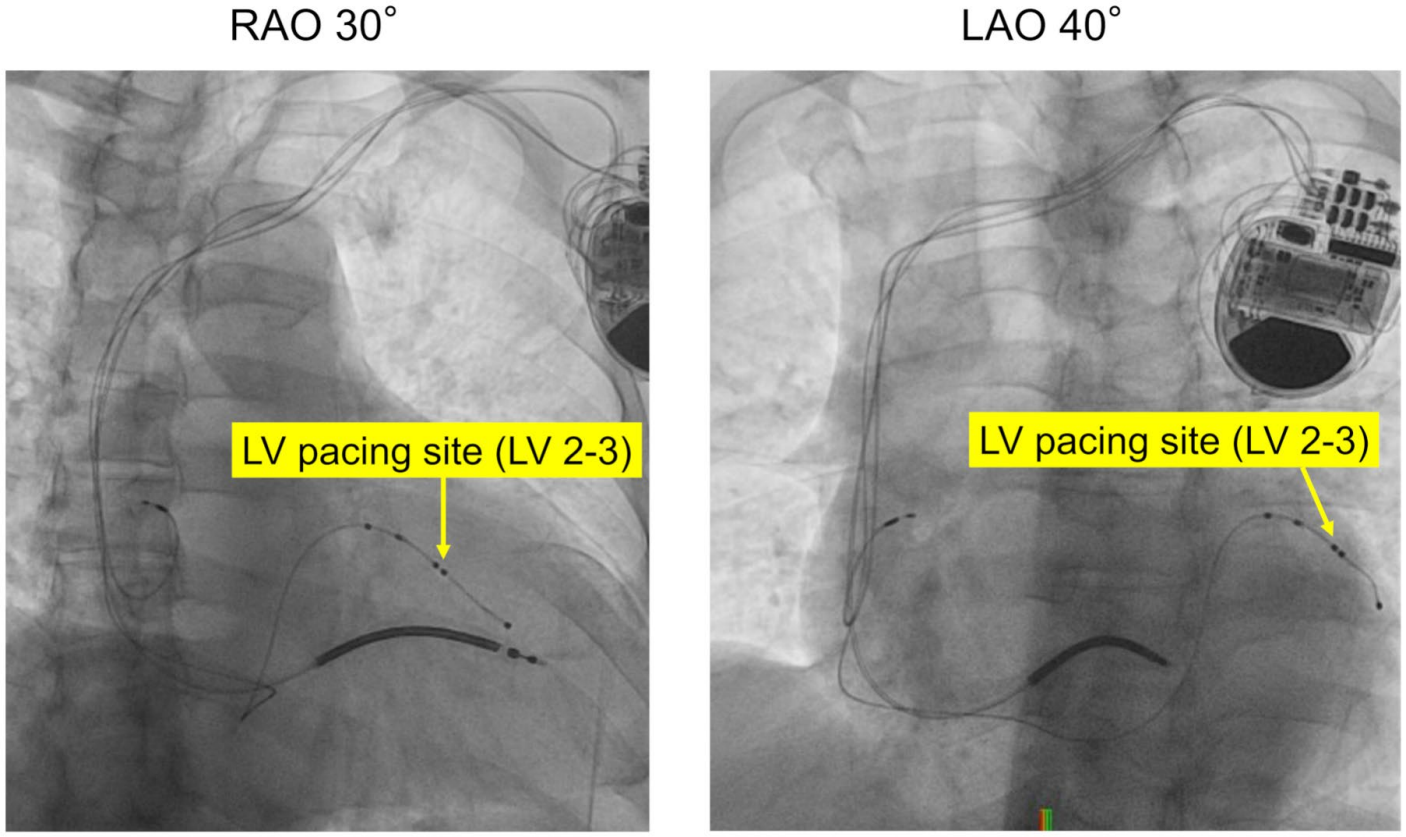
Lead positions after cardiac resynchronization therapy with defibrillator upgrade. The atrial lead was positioned at the high atrial septum, and the left ventricular (LV) lead was positioned in the anterolateral coronary sinus branch. The LV mid site (LV 2–3) showed the greatest electrical delay, with a QLV of 100 ms, and was selected as the LV pacing site (yellow arrow). LAO, left anterior oblique; LV, left ventricular; RAO, right anterior oblique.

At 8 months after CRT‐D upgrade, the patient's symptoms improved to NYHA functional Class II. Brain natriuretic peptide decreased to 73 pg/mL and serum creatinine to 1.43 mg/dL. Although transient episodes of paroxysmal AF were observed shortly after discharge, overall AF control remained satisfactory (Figure 4A). Echocardiography demonstrated an LVEF of 16% and an LVEDVI of 142 mL/m^2^ and an LVESVI of 121 mL/m^2^, indicating that the patient was not a volume responder. However, LAVI decreased to 57.5 mL/m^2^, accompanied by improvement in mitral regurgitation. Left atrial strain analysis demonstrated markedly reduced reservoir and contractile strain during sinus rhythm, indicating severe atrial dysfunction. In contrast, BBP improved left atrial contractile function (Figure 4B).

**FIGURE 4:**
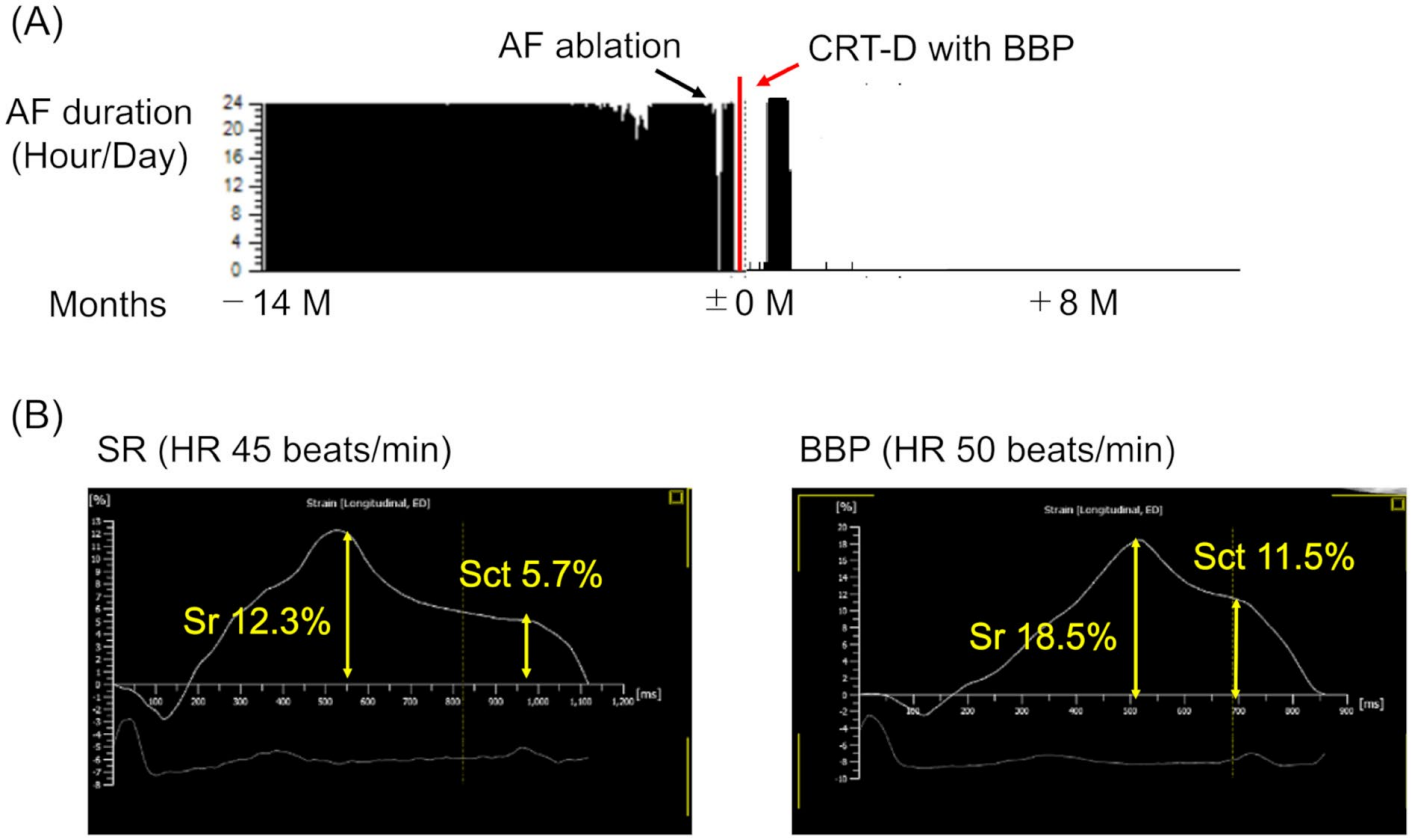
(A) Atrial fibrillation (AF) burden before and after cardiac resynchronization therapy with defibrillator (CRT‐D) upgrade. The vertical axis represents AF duration per day, and the horizontal axis represents time with the CRT‐D upgrade procedure defined as Month 0. Although AF control remained difficult despite AF ablation (black arrow), AF control improved markedly after CRT‐D upgrade with Bachmann's bundle pacing (BBP) (red arrow). (B) During sinus bradycardia (heart rate, 45 beats/min), reservoir strain was 12.3% and contractile strain was 5.7%, indicating severely impaired atrial function. During BBP (heart rate, 50 beats/min), reservoir strain improved to 18.5% and contractile strain to 11.5%, demonstrating improved left atrial contractile function. AF, atrial fibrillation; BBP, Bachmann's bundle pacing; CRT‐D, cardiac resynchronization therapy with defibrillator; HR, heart rate; Sct, contractile strain; Sr, reservoir strain; SR, sinus rhythm.

In this case, BBP performed during CRT was associated with a high LV‐only pacing percentage, favorable atrial remodeling, and effective AF control despite limited improvement in LV function. Furthermore, in this patient with first‐degree AV block, BBP allowed atrial pacing without prolonging AV conduction time, thereby maintaining a high LV‐only pacing percentage. Optimization of AV timing through pacing may also have improved atrial filling and emptying and reduced left atrial volume loading, potentially contributing to the functional response [4, 5].

Given the presence of NICD, LOT‐CRT could have been considered; however, marked atrial and ventricular dilatation was considered a potential factor reducing the likelihood of successful left bundle branch area pacing. No other coronary sinus branches of suitable size were available, whereas the anterolateral branch allowed LV pacing at a site with substantial electrical delay. The QRS morphology was suggestive of left anterior fascicular block, and the relatively long QLV supported delayed activation of the anterolateral region. Furthermore, the LV lead was positioned remote from and opposite to the RV lead, potentially favoring ventricular resynchronization. The AdaptivCRT algorithm was therefore used to promote LV‐only pacing while preserving intrinsic RV activation.

This study has several limitations. First, AV conduction time during RAA pacing was not measured at the time of CRT‐D upgrade. Second, we did not compare AV conduction time or LV‐only pacing percentage with low atrial septal pacing. Although low atrial septal pacing may also maintain a high LV‐only pacing percentage due to its anatomical proximity to the AV node, BBP has been reported to be more effective in suppressing AF than other atrial septal pacing sites [3], supporting the clinical relevance of BBP selection in this case. Third, the efficacy of the Adaptive CRT algorithm in this patient remains uncertain. The AdaptResponse trial, a multicenter, prospective, randomized controlled trial, enrolled patients with LBBB [2], whereas the present patient did not have typical LBBB but rather NICD. Therefore, it remains unclear whether the favorable clinical outcomes observed in this patient were attributable to the high percentage of LV‐only pacing achieved with the Adaptive CRT algorithm, to the effects of BBP itself, or to a combination of both. Finally, this single case cannot establish the efficacy of BBP in CRT, warranting further multicenter studies.

In conclusion, BBP in CRT recipients with first‐degree AV block may improve LV‐only pacing percentage, suppress AF, and potentially enhance CRT response.

## Funding

The authors have nothing to report.

## Ethics Statement

Ethical approval was waived by the Institutional Review Board of Komaki City Hospital because this report describes a single case.

## Consent

Written informed consent was obtained from the patient for publication of this case and accompanying images.

## Conflicts of Interest

The authors declare no conflicts of interest.

## Data Availability

The data supporting the findings of this study are available from the corresponding author upon reasonable request.
